# The Key Role of Genomics in Modern Vaccine and Drug Design for Emerging Infectious Diseases

**DOI:** 10.1371/journal.pgen.1000612

**Published:** 2009-10-26

**Authors:** Kate L. Seib, Gordon Dougan, Rino Rappuoli

**Affiliations:** 1 Novartis Vaccines and Diagnostics, Siena, Italy; 2 The Wellcome Trust Sanger Institute, The Wellcome Trust Genome Campus, Hinxton, Cambridge, United Kingdom; University of California San Diego and The Scripps Research Institute, United States of America

## Abstract

It can be argued that the arrival of the “genomics era” has significantly shifted the paradigm of vaccine and therapeutics development from microbiological to sequence-based approaches. Genome sequences provide a previously unattainable route to investigate the mechanisms that underpin pathogenesis. Genomics, transcriptomics, metabolomics, structural genomics, proteomics, and immunomics are being exploited to perfect the identification of targets, to design new vaccines and drugs, and to predict their effects in patients. Furthermore, human genomics and related studies are providing insights into aspects of host biology that are important in infectious disease. This ever-growing body of genomic data and new genome-based approaches will play a critical role in the future to enable timely development of vaccines and therapeutics to control emerging infectious diseases.

By controlling debilitating and often-lethal infectious diseases, vaccines and antibiotics have had an enormous impact on world health. Now, with the arrival of the “genomics era,” a paradigm shift is occurring in the development of vaccines—and potentially also in the development of antibiotics—that is providing fresh impetus to this field. The world is still faced with a huge burden of infection, however, by classic pathogens (e.g., typhoid, measles), recently discovered causes of disease (e.g., *Helicobacter pylori* and hepatitis C virus [HCV]), and emerging infectious diseases (EIDs, e.g., H1N1 swine flu and severe acute respiratory syndrome coronavirus [SARS-CoV]). In addition, variant forms of previously identified infectious diseases are reemerging (e.g., *Streptococcus pyogenes*, also known as group A streptococcus [GAS], and dengue fever), along with antibiotic-resistant forms of microbes (e.g., methicillin-resistant *Staphylococcus aureus* [MRSA] and *Mycobacterium tuberculosis*) [1],[2] (for a list of EIDs see http://www3.niaid.nih.gov/topics/emerging/list.htm). The World Health Organization (WHO) estimates that we can expect at least one such new pathogen to appear every year.

The fact that an infectious disease has emerged or reemerged indicates immune naïvety in the infected population, or altered virulence potential or an increase in antibiotic/antiviral resistance in the pathogen population. The rapid development of vaccines and therapeutics that target these pathogens is therefore essential to limit their spread. Traditional empirical approaches that screen for vaccines or drugs a few candidates at a time are time-consuming and have often proven insufficient to control many EIDs, particularly when the causative pathogens are antigenically diverse (e.g., HIV), cannot be cultivated in the laboratory (e.g., HCV), lack suitable animal models of infection (e.g., *Neisseria* spp.), have complex mechanisms of pathogenesis (e.g., retroviruses), and/or are controlled by mucosal or T cell–dependent immune responses rather than humoral immune responses (e.g., *Shigella* spp., *M. tuberculosis*) [3]. For many EIDs, the wealth of information emerging in the genome era has already had a significant impact on the way we approach vaccine and therapeutic development. For EIDs that appear in the near future, genomics will be in the first line of defense in terms of antigen identification, diagnostic development, and functional characterization.

Since the completion of the genome sequence of *Haemophilus influenzae*—the first finished bacterial genome sequence—in 1995 [4], advances in sequencing technology and bioinformatics have produced an exponential growth of genome sequence information. At least one genome sequence is now available for each major human pathogen. As of October 2009, over 1,000 bacterial genomes were “completed” (i.e., closed genomes and whole genome shotgun sequences) and more than 1,000 were ongoing; over 3,000 viral genomes were completed (http://www.genomesonline.org/gold.cgi, http://www.ncbi.nlm.nih.gov/genomes/MICROBES/microbial_taxtree.html, http://cmr.jcvi.org/tigr-scripts/CMR/shared/Genomes.cgi). For a bacterial pathogen, which may have more than 4,000 genes, the genome sequence provides the complete genetic repertoire of antigens or drug targets from which novel candidates can be identified. For viral pathogens that may possess fewer than 10 genes, genomics can be used to define the variability that may exist between isolates. Host genetic factors also play a role in infectious disease [5],[6], however, and the availability of “complete” human genome sequences, as well as large-scale human genome projects (see http://www.1000genomes.org/), are valuable resources. Hence, the sequences of both pathogen and host genomes can facilitate identification of a growing number of potential vaccine and drug targets (Figure 1). It is estimated that 10–100 times more candidates can be identified in one to two years using genomics-based approaches than can be identified by conventional methods in the same time frame. Furthermore, genomics-based vaccine projects have substantially increased our understanding of microbial physiology, epidemiology, pathogenesis, and protein functions (see Box 1).

**Figure 1:**
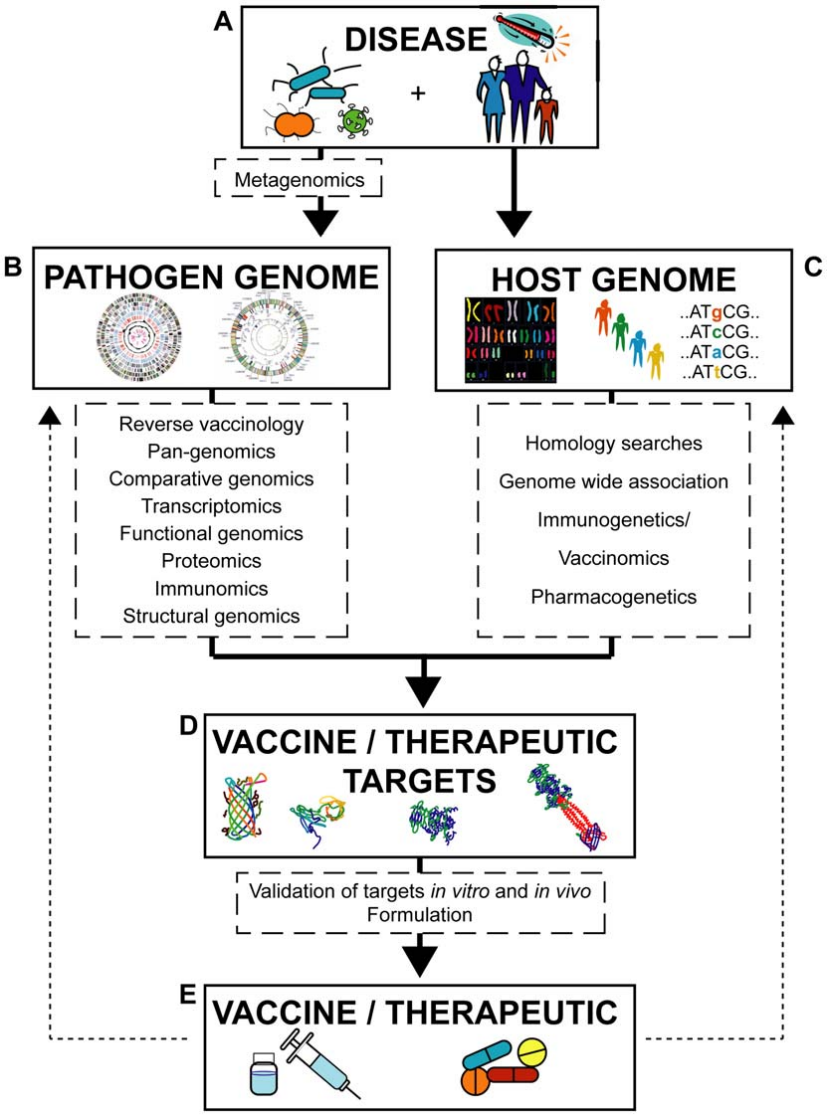
Genomics-based approaches used in the control of EIDs from the outbreak of a disease to the development of a vaccine or drug. (A) The causative agent of a disease may first be identified from patient samples by using metagenomics. (B) Vaccine and therapeutic targets can be identified from the pathogen genome using a variety of screening approaches that focus on the genome, transcriptome, proteome, immunome or structural genome. (C) The human genome can be screened to avoid homologies or similarities with pathogen vaccine and therapeutic targets, or to identify new targets. (D) Once candidate vaccine and therapeutic targets have been identified they must be shown to provide protection against disease and to be safe for use in patients. (E) The clinically tested vaccine or therapeutic can then be licensed for use. The clinical responses of a vaccine and/or therapeutic can be analyzed using human genome based studies (dotted arrows). The pathogen genome can also be used to analyze mutants that are able to evade the immune system in vaccinated subjects or organisms that develop antibiotic resistance. Examples of the approaches indicated are given in Table 1.

**Box 1: Reverse Vaccinology Drives the Discovery of New Protein Functions**

Reverse vaccinology involves the in silico screening of the entire genome of a pathogen to find genes that encode proteins with the attributes of good vaccine targets, using either the genome of a single pathogenic isolate or the pan-genome (the genomic information from several isolates) of a pathogenic species.

**Pili in pathogenic streptococci play a key role in virulence and are promising vaccine candidates**

The identification of pili (long filamentous structures that extend from the bacterial surface) in the main pathogenic strains of streptococci is a good example of how genomics can lead to the discovery of protein functions and increased understanding of host–pathogen interactions. The pili of gram-negative bacteria are well-described virulence factors. Little was known, however, about pili in gram-positive bacteria before the sequencing and analysis of the genomes of *S. pyogenes*, *S. agalactiae*, and *S. pneumoniae* (reviewed in [72]).

During analysis of eight *S. agalactiae* genome sequences, three protective antigens identified by pan-genomic reverse vaccinology [20] were found to contain LPXTG motifs typical of cell wall-anchored proteins and seen to assemble into pili [73]. Further bioinformatics analysis revealed three independent loci that encode structurally distinct pilus types, each of which contains two surface-exposed antigens capable of eliciting protective immunity in mice [75]. Because of the limited variability of *S. agalactiae* pili, it has been suggested that a combination of only three pilin subunits could lead to broad protective immunity [74].

Following the identification of *S. agalactiae* pili, typical pilus regions were identified in the available *S. pyogenes* genomes based on the presence of genes encoding LPXTG-containing proteins. In addition, a combination of recombinant pilus proteins was shown to confer protection in mice against mucosal challenge with virulent *S. pyogenes* isolates [75]. Falugi and colleagues have since found that *S. pyogenes* pili are encoded by nine different gene clusters, and they estimate that a vaccine comprising a combination of 12 backbone variants could provide protection against over 90% of circulating *S. pyogenes* strains [76].

The availability of multiple complete genome sequences for *S. pneumoniae*, and the increased understanding of pilus proteins in other pathogenic streptococci, led to the discovery of two pilus “islands” that encode proteins that play a role in adherence to lung epithelial cells and colonization in a murine model of infection, where they elicit host inflammatory responses [77],[78]. In addition, the pilus subunits confer protection in passive and active immunization models [79]. The presence of pili that contain protective antigens in all three principal streptococcal pathogens indicates that these structures play an important role in virulence.

**Reverse vaccinology leads to identification of the fHBP and its role in meningococcal species specificity**

Serogroup B *N. meningitidis* (MenB) strains are responsible for the majority of meningococcal disease in the developed world, yet there is no comprehensive MenB vaccine available. Screening of the MenB genome for vaccine candidates by using reverse vaccinology led to the discovery of the meningococcal factor H-binding protein (fHBP) [15], which was recently suggested to play an important role in the species specificity of *N. meningitidis* [80]. fHBP is a component of the Novartis multivalent MenB vaccine that entered Phase III clinical testing in 2008 [16],[17] and is also under investigation by Wyeth Vaccines (designated LP2086) [81] and other groups [82]. Initially identified as the genome-derived *Neisseria* antigen 1870 (GNA1870), a *Neisseria*-specific putative surface lipoprotein of unknown function, fHBP was renamed because of its ability to bind complement factor H (fH), a molecule that down-regulates activation of the complement alternative pathway. Hence, binding of fH to the surface of *Neisseria* allows the pathogen to evade complement-mediated killing by the innate immune system [83]. fHBP is expressed by all *N. meningitidis* strains studied [84]. It induces high levels of bactericidal antibodies in mice [16] and is important for survival of bacteria in human serum and blood [83],[85],[86]. The discovery that binding of fH to *N. meningitidis* is specific for human fH, and that human fH alone is able to down-regulate complement activation and bactericidal activity leading to increased bacterial survival has significant implications for the study of this organism [80]. The administration of human fH to infant rats challenged with MenB led to a greater than 10-fold increase in survival of bacteria [80], providing an important insight into host–pathogen interactions that may lead to the development of new animal models of infection.

From the outbreak of a disease, metagenomics (the study of all the genetic material recovered directly from a sample) can be applied to diseased human samples to aid the rapid identification of the causative agent [7],[8]. Once the complete genome sequence of the organism is available, high-throughput approaches can be used to screen for target molecules, as outlined below and in Table 1 [9],[10]. Screening approaches vary depending on the nature of the pathogen but are based on several accepted principles and key requirements of vaccines and therapeutics, including the need for targets to be (i) expressed and accessible to the host immune system, or to a therapeutic agent, during human disease; (ii) genetically conserved; (iii) important for survival or pathogenesis; and (iv) free of measurable homology or similarity to host factors. Although many of the approaches described here focus on vaccine development, which involves screening of candidates for immunogenicity, they are largely applicable to drug development by altering the selection criteria used and screening candidates against compound libraries [11]–[13].

**Table 1:** Approaches to identify vaccine and/or drug targets against EIDs in the genomic era.

Approach	Methods Used	Limitations of Method	Example
			Organism	Disease
**Genomics/reverse vaccinology:** Analysis of the genetic material of an organism in order to identify the repertoire of protein antigens/drug targets the organism has the potential to express.	Bioinformatics screening of the genome sequence to identify ORFs predicted to be exposed on the surface of the pathogen or secreted, expression of recombinant proteins, generation of antibodies in mice to confirm surface exposure, and bactericidal activity [14].	Prediction algorithms need to be validated. Non-protein antigens including polysaccharides or glycolipids, and post-translational modifications cannot be identified. High-throughput cloning and protein expression is required.	Serogroup B *N. meningitidis* [15],[16]	Major cause of septicemia and meningitis in the developed world.
**Pan-genomics:** Analysis of the genetic material of several organisms of a single species to identify conserved antigens/targets and ensure the chosen target covers the diversity of the organism.	Similar to above, but ORFs are chosen by screening of multiple genomes with either direct sequencing or comparative genome hybridization [18].	Sequences of multiple isolates of a species are required. Similar limitations as described above.	*S. agalactiae* [20]	Leading cause of neonatal bacterial sepsis, pneumonia, and meningitis in the US and Europe.
**Comparative genomics:** Analysis of the genetic material of several individuals of a single species, to identify antigens/targets that are present in pathogenic strains but absent in commensal strains, and thus important for disease.	Similar to pangenomics, but ORFs are chosen by screening of genomes from multiple strains of pathogenic and commensal strains of a species [18],[21].	Similar limitations as for the above two approaches.	*E. coli* [22]	Major cause of mild to severe diarrhea, hemolytic-uremic syndrome, and urinary tract infections.
**Transcriptomics:** Analysis of the set of RNA transcripts expressed by an organism under a specified condition.	Gene expression is evaluated in vitro or in vivo using DNA microarrays or cDNA sequencing [24].	There is no direct correlation between the levels of mRNA and protein. In vivo studies require relatively large amounts of mRNA.	*V. cholerae* [26]	Causes diseases ranging from self-limiting to severe, life-threatening diarrhea, wound infections, and sepsis.
**Functional genomics:** Analysis of the role of genes and proteins in order to identify genes required for survival under specific conditions.	Genes that are functionally essential in specific conditions in vitro or in vivo are determined by gene inhibition followed by screening of mutants in animal models or cell culture to identify attenuated clones [87].	Genetic tools, acceptance of transposons, and natural competence of the pathogen are required.	*H. pylori* [32]	Major cause of duodenal and gastric ulcers and stomach cancer as a result of chronic low-level inflammation of the stomach lining.
**Proteomics:** Analysis of the set of proteins expressed by an organism under a specified condition and/or in specific cellular locations (e.g., on the cell surface).	2D-PAGE, MS, and chromatographic techniques to identify proteins from whole cells, fractionated samples, or the cell surface [34].	Proteins with low abundance and/or solubility and proteins that are only expressed in vivo may not be identified.	*S. pyogenes* [36]	Cause of a range of diseases from mild pharyngitis to severe toxic shock syndrome, necrotizing fasciitis, and rheumatic fever.
**Immunomics:** Analysis of the subset of proteins/epitopes that interact with the host immune system.	Analysis of seroreactive proteins, using 2D-PAGE, phage display libraries, or protein microarrays, probed with host sera [38]. Bioinformatics prediction of B cell and T cell epitopes [37].	Potential bias against sequences that cannot be displayed. Large conformational epitopes made up of noncontiguous amino acids may not be detected. Prediction of B cell epitopes is difficult due to the need to identify conformational epitopes.	*S. aureus* [39]	Cause of wound infections. Has emerged as a significant opportunistic pathogen due to antibiotic resistance.
**Structural genomics:** Analysis of the three-dimensional structure of an organism's proteins and how they interact with antibodies or therapeutics.	NMR or crystallography to determine the structure of proteins in the presence/absence of antibodies or therapeutics [51].	Poor understanding of determinants of immunogenicity, immunodominance, and structure-function relationships.	HIV [53]	Causative agent of AIDS.
**Vaccinomics/immunogenetics pharmacogenetics:** Analysis of how the human immune system responds to a vaccine or drug.	Investigation of genetic heterogeneity/polymorphisms in the host, at the individual or population level, that may alter immune responses to vaccines [68] or metabolism of therapeutics [71].	Ethical issues of “personalized” medicine. Immense diversity of the human genome and, in particular, in the human immune response.	Mumps virus [69]	Cause of disease ranging from self-limiting parotid inflammation to epididymo-orchitis, meningitis, and encephalitis.

## Reverse Vaccinology, Pan-genomics, and Comparative Genomics

The idea behind reverse vaccinology is to screen an entire pathogen genome to find genes that encode proteins with the attributes of good vaccine targets, such as, for example, bacterial surface associated proteins [14]. These proteins can then undergo normal laboratory evaluation for immunogenicity. The *Neisseria meningitidis* serogroup B (MenB) reverse vaccinology project provides the “proof of concept” for this type of approach. This project identified more novel vaccine candidates in 18 months than had been discovered in 40 years of conventional vaccinology [15]. Analysis of the genome sequence of the virulent MenB strain MC58 found 2,158 predicted open reading frames (ORFs); these were screened using bioinformatics tools to identify 570 ORFs that were predicted to encode surface-exposed or secreted proteins that might be accessible to the immune system [15]. Antigen screening continued on the basis of several criteria: the ability of antigens to be expressed in *Escherichia coli* as recombinant proteins (350 candidates); confirmation by ELISA and flow cytometry that the antigen is exposed on the cell surface (91 candidates); the ability of induced antibodies to elicit killing, as measured by serum bactericidal assay and/or passive protection in infant rat assays (28 candidates); and screening of a panel of diverse meningococcal isolates to determine whether the antigens are conserved. This approach resulted in the development of a multi-component recombinant MenB vaccine that entered Phase III clinical trials in 2008 [16],[17].

As multiple genome sequences become available for a single species, the concept of pan-genomic reverse vaccinology is emerging as a powerful tool to identify vaccine candidates in antigenically diverse species [18]. Pan-genomics aims to identify the full complement of genes in a species, based on the superset of genes in several strains of the same species. Analysis of the genome sequences of eight *Streptococcus agalactiae* (also known as group B streptococcus) strains revealed substantial genetic heterogeneity and the extended gene repertoire of the species [19]. Screening found a total of 589 genes predicted to encode surface-exposed or secreted proteins in the *S. agalactiae* pan-genome (396 from the “core genome”—genes conserved in all strains—and 193 from the “dispensable genome”—genes that are present in two or more strains and are hence considered dispensable for survival). Based on further screening of this pool of candidates, including the ability of recombinant proteins to provide protection when used to immunize animals, a combination of four antigens—only one of which is in the core genome—was selected and shown to confer protection against a panel of *S. agalactiae* strains [20].

Whereas genome sequencing projects have typically focused on pathogenic organisms, comparison of the genomes of pathogenic and nonpathogenic strains allows vaccine and drug targets to be identified on the basis of proteins that are specifically involved in pathogenesis [21]. Comparative studies of up to 17 commensal and pathogenic *E. coli* genomes identified genes unique to certain pathogenic strains that are largely absent in commensal strains. This filter decreases the pool of targets to be screened and potentially limits any detrimental effects of therapeutics on the composition of the commensal flora [22].

New sequencing technologies will also open up opportunities for monitoring pathogen vaccine escape by screening for evidence of immune selection in the genomes of pathogen populations before and after vaccine selection. By deep-sequencing of bacterial and viral populations it will be possible to identify antigens under immune selection by monitoring the clustering of single nucleotide polymorphisms (SNPs) and other mutations that affect protein sequence. This approach has already been used to search for evidence of antigenic variation/selection in populations of *Salmonella enterica* serovar Typhi [23], where variation is extremely limited. Similar sequencing strategies could be applied to populations of bacteria taken before or after a vaccine trial in a particular geographical region.

## Beyond Genomics: Other -Omics Approaches to Study Pathogens

Pathogen genes that are up-regulated during infection and/or essential for microorganism survival or pathogenesis can be identified by using transcriptomics, i.e., the analysis of a near complete set of RNA transcripts expressed by the pathogen under a specified condition. Comprehensive DNA-based microarray chips (probed with cDNA generated from RNA by reverse transcription) [24] and ultra-high-throughput sequencing technologies that allow rapid sequencing and direct quantification of cDNA [25] enable the transcriptome of a pathogen to be characterized and particular types of gene product to be identified. For example, genes involved in the hyperinfectious state of *Vibrio cholerae*, which appears after passage through the human gastrointestinal tract, were identified through a comparison of the transcriptome of bacteria isolated directly from stool samples of cholera patients with that of *V. cholerae* grown in vitro [26]. Similarly, analysis of the transcription profile of *M. tuberculosis* during early infection in immune-competent (BALB/c) and severe combined immunodeficient (SCID) mice revealed a set of 67 genes activated exclusively in response to the host immune system [27].

Functional genomics—linking genotype, through transcriptomics and proteomics, to phenotype—has been applied to many pathogens to identify genes essential to survival or virulence that may be valid vaccine candidates. DNA microarrays can be used to screen comprehensive libraries of pathogen mutants, by comparing bacterial isolates from before and after passage through animal models or exposure to compound libraries to identify attenuated clones [28]–[30]. For example, these methods have been used to identify 65 novel MenB genes that are required for the pathogen to cause septicemia in infant rats [31], 47 genes essential for *H. pylori* gastric colonization of the gerbil [32], and genes contributing to *M. tuberculosis* persistence in the host [33].

Analysis of a pathogen's proteome (the near complete set of proteins expressed under a specified condition) to reveal potential vaccine and drug candidates can add significant value to in silico approaches [34]. High-throughput proteomic analyses can be performed by using mass spectrometry (MS), chromatographic techniques, and protein microarrays [35]. A novel proteome-based approach has been applied to identify the surface proteins of GAS by making use of proteolytic enzymes to “shave” the bacterial surface, releasing exposed proteins and partially exposed peptides. Seventeen surface proteins of a virulent GAS strain were identified in this way by using MS and genome sequence analysis. Their location on the pathogen surface was confirmed by flow cytometry, and one of them provided protective immunity in a mouse model of the disease [36].

The proteome of a pathogen can also be screened to identify the immunome (the near complete set of pathogen proteins or epitopes that interact with the host immune system) using in vitro or in silico techniques [37],[38]. In vitro identification and screening of the immunome are based on the idea that antibodies present in serum from a host, which has been exposed to a pathogen, represent a molecular “imprint” of the pathogen's immunogenic proteins and can be used to identify vaccine candidates. As such, several techniques have been developed to allow the high-throughput display of pathogen proteins, and the subsequent screening for proteins that interact with antibodies in sera. Immunogenic surface proteins of several organisms have been identified, including *S. aureus* using 2D-PAGE, membrane blotting, and MS [39]; *S. agalactiae*, *S. pyogenes*, and *Streptococcus pneumoniae* using phage- or *E. coli*-based comprehensive genomic peptide expression libraries [38],[40]; and *Francisella tularensis* (the causative agent of tularemia or rabbit fever) [41] and *V. cholerae* using protein microarray chips [42]. Protein microarrays, in which proteins from the pathogen are spotted onto a microarray chip, can also be used to characterize protein–drug interactions, as well as other protein–protein, protein–nucleic acid, ligand–receptor, and enzyme–substrate interactions [43].

The ability to predict in silico which pathogen epitopes will be recognized by B cells or T cells has greatly improved in recent years [44]. Large-scale screening of pathogens including HIV, *Bacillus anthracis*, *M. tuberculosis*, *F. tularensis*, *Yersinia pestis* (the causative agent of bubonic plague), flaviviruses, and influenza for B cell and T cell epitopes is currently underway [45],[46]. Although epitope prediction is not foolproof, it can serve as a guide for further biological evaluation. T cell epitopes are presented by MHC/HLA proteins on the surface of antigen-presenting cells, which vary considerably between hosts, complicating the task of functional epitope prediction. Additionally, B cell epitopes can be both linear and conformational. The ultimate aim of researchers in this field of study would be to engineer a single peptide that represents defined epitope combinations from a protein or organism, enabling the genetic variability of both pathogen and host to be overcome [44].

Structural genomics—the study of the three-dimensional structures of the proteins produced by a species—is increasingly being applied to vaccine and drug development as a result of the explosion of genome and proteome data, and continuing improvements in the fields of protein expression, purification, and structural determination [47]. The structure-based design of antiviral therapeutics has led to the development of drugs directed at the active sites of the HIV-1 protease [48] and influenza neuraminidase [49]. More than 45,000 high-resolution protein structures are available in public databases (see http://www.wwpdb.org/stats.html), and several initiatives have been established to pursue high-throughput characterization of protein structures on a genome-wide scale [50], focusing on determining and understanding the structural basis of immune-dominant and immune-recessive antigens as well as protein active sites and potential drug-binding sites [51],[52]. For example, structural characterization of the HIV envelope proteins gp120 and gp41 has revealed mechanisms used by the virus to evade host antibody responses, many of which involve hypervariability in immunodominant epitopes [53],[54]. Based on this information, immune refocusing (e.g., by retargeted glycosylation, deletion, and/or substitution of amino acids) has been used to dampen the response to variable immunodominant epitopes of the envelope glycoprotein gp160, enabling the host to respond to previously subdominant epitopes [55]. High-throughput modification of proteins and their screening for immunogenicity and interaction with antimicrobials is predicted to become more common as techniques evolve [51].

## The Contribution of Human Genomics

When designing new vaccines, one important consideration is the risk that the vaccine might generate “self” immune reactions against host epitopes; immune responses against a pathogen antigen can cross-react with host antigens if homologies exist in the primary amino acid sequence or structure, potentially leading to damage to the host tissue [56]. Drugs aimed at pathogen targets could also theoretically target similar host molecules. The availability of the human genome sequence combined with methods for predicting B cell and T cell epitopes will facilitate screening for the presence of homologies between candidate microbial vaccine antigens and proteins in humans, enabling issues of autoimmunity and cross-reactivity to be tackled [57]. As such, vaccine or drug targets identified using methods based on pathogen genomics should be screened for homology or similarity to human proteins in silico, using programs such as BLAST (Basic Local Alignment Search Tool; http://blast.ncbi.nlm.nih.gov/Blast.cgi) to query human genome databases. Interestingly, analysis of 30 viral genomes revealed that around 90% of viral pentapeptides, which could be components of epitopes, are identical to human peptides [58]. There is little homology, however, between validated immunogenic disease-associated peptides/epitopes and host peptides [57],[59], suggesting that screening approaches that include prediction of immunogenicity could improve the pool of target candidates.

It is important to keep in mind that we do not fully understand how self-tolerance is broken, so we currently have no perfect way of predicting all potential autoimmune triggers that could be associated with vaccination. While many links have been made between autoimmune disease and vaccination, they have been confirmed in only a small number of cases (reviewed in [60]). For example, treatment-resistant Lyme arthritis is associated in certain patients with immune reactivity to the outer surface protein A (OspA) of the causative agent of Lyme disease, *Borrelia burgdorferi*, and an OspA epitope (OspA165–173) has homology to the human lymphocyte function-associated antigen (hLFA)-1αL [61]. As a result, the OspA-based Lyme disease vaccine (LYMErix) was taken off the market in 2002, but a recombinant OspA lacking the potentially autoreactive T cell epitope has been proposed as a replacement vaccine [62].

Rather than targeting drugs to pathogen enzymes, an alternative approach has focused on targeting the host-cell proteins that are exploited by pathogens for replication and survival. The use of techniques including microarray-based analysis of virus-induced host gene expression has revealed several possible targets [63],[64]. The cholesterol-lowering drugs statins, for example, have an anti-HIV effect that is believed to be mediated by preventing activation of the host protein Rho, which is activated by the HIV envelope protein and required for virus entry to the cell [65]. Furthermore, such studies can improve our understanding of the host immune responses that protect against a pathogen (i.e., innate, antibody, Th1, or Th2 responses), which will aid the selection of appropriate vaccine adjuvants. For example, induction of interferon signaling early in infection may be critical to confer protection against SARS-CoV, as determined from functional genomic studies of early host responses to SARS-CoV infection in the lungs of macaques [66].

Many of the genes of the human immune system are highly polymorphic, which enables the population as a whole to generate sufficient immunological diversity to combat EIDs. This variation also impacts on the outcome of vaccination and treatment. The International HapMap Project has identified over 3.1 million SNPs in 270 individuals [67] and the 1000 Genomes Project aims to identify even more genetic variants. The field of vaccinomics (also called immunogenetics) investigates heterogeneity in host genetic markers that results in variations in vaccine-induced immune responses, with the aim of predicting and minimizing vaccine failures or adverse events [68]. For example, polymorphisms of HLA and immunoregulatory cytokine receptor genes are associated with variable outcomes of vaccination against mumps [69]. Similarly, pharmacogenetics, which investigates genetic differences in the way individuals metabolize therapeutics, has found that human variability in the speed of metabolism of the common first-line tuberculosis drug isoniazid is associated with genetic variants, including SNPs, in the gene encoding arylamine N-acetyltransferase (NAT2) [70],[71]. The ability to predict an individual's response to a vaccine or drug, may eventually allow physicians to determine whether a patient is genetically susceptible to a disease, the possible adverse effects of a vaccine or drug, and the appropriate schedule or dose to use.

## Challenges for the Future

We predict that genomics will greatly aid the control of EIDs because of the increased efficiency with which vaccine and therapeutic targets can be identified using the genome-based approaches described above. Furthermore, we anticipate the continual refinement and development of novel genome-based approaches as sequencing becomes faster and more affordable. Several challenges remain, however, in the identification of these targets and in the processes needed to bring a new vaccine or drug to the market. Understanding the molecular nature of epitopes, the mechanisms of action of adjuvants, and T cell and mucosal immunity are key priorities to be tackled in the coming years [3]. These issues can be addressed by improved structural studies of antigen epitopes and the compilation of databases containing information on structure, immunogenicity, and in silico B cell and T cell epitope predictions. Genome-based development of effective vaccines and therapeutics is still largely dependent on the availability of valid models to measure efficacy and protection against disease; however, the increased understanding of microbial pathogenesis that is emerging from genomics should greatly aid in this respect. Likewise, the continued development of animal models with knockout and allele-specific mutations in key components of the immune response will greatly increase understanding of the type of immune response needed to control disease and the ways in which the immune system can be programmed to protect the host against disease. Unfortunately, the stepwise series of prelicensure clinical trials (Phase I, II, and III) that are required to document the safety, immunogenicity, and efficacy of a vaccine are still highly time-consuming and costly. We can only hope that the increasingly “smart” identification and design of targets, and the fresh impetuous given to the fields of vaccine and drug development by the arrival of genomics, will enable increased success of those vaccines and drugs that do make it into clinical development.
